# Online mental health interventions designed for students in higher education: A user-centered perspective

**DOI:** 10.1016/j.invent.2021.100468

**Published:** 2021-10-09

**Authors:** Olugbenga Oti, Ian Pitt

**Affiliations:** School of Computer Science & IT, University College Cork, Ireland

**Keywords:** User-centered design, E-mental health, Mental wellbeing, Higher education

## Abstract

**Background:**

Digital mental health interventions have been posited as a way of reducing the burden on mental health services in higher education institutions. However, low adherence and high attrition rates present a challenge that limits the effectiveness of these interventions. User-centered design has been proposed as a suitable approach in improving the adherence of users to these interventions.

**Objective:**

The objective of this scoping review was to examine digital mental health interventions that have been designed specifically for students in higher education. It aimed to summarize the published literature on digital mental health interventions which take a user-centered approach in developing interventions for students in higher education.

**Methods:**

A scoping review of peer-reviewed research papers from the following electronic databases was conducted: Embase, ACM digital library, Web of Science, IEEE Explore, SCOPUS, EBSCO Host (including APA PyscInfo, CINAHL PLUS, APA PsycArticles, Medline), PubMed and Google Scholar. Databases were searched from inception until 13 Jan and 14 Jan 2021. Of the 755 articles that were identified, 57 articles were selected for full review. 34 articles were excluded for not matching the inclusion criteria.

**Results:**

23 studies were included in this review. The included interventions targeted various areas of mental health including depression, anxiety, overall wellbeing, and mental health awareness. The interventions were commonly delivered through mobile apps, web-based apps, and desktop apps. In addition, we explore design methodologies applied in the development of the interventions: we note significant stakeholder engagement in the studies, the inclusion of multiple stakeholder types (students, health care professionals, university staff, and young people in the general population), and limited use of design frameworks. Finally, in exploring user engagement, attrition rates and user acceptance, we find that most of the studies have not progressed enough (i.e., at pilot/prototype stages of development) to determine the impact of design methodologies on the success of these interventions.

**Conclusion:**

Our review revealed a need for further research on the impact of user-centered design practices on the success of digital mental health interventions in this population. Further, we provide recommendations that researchers/designers in this field of research should take into consideration when designing online mental health interventions for students in higher education. Some of the recommendations include: add personalization; improve user interfaces; take adequate steps to ensure anonymity/privacy/security; include peer engagement; and include access to mental health professionals.

## Highlights

• Mental health difficulties like depression and anxiety are known to disproportionately affect the youth population.
• Mental health support services are often burdened by a large number of students seeking help.
• Online mental health interventions have been introduced to bridge the gap in providing mental health support to students.
• Improving user experience is one of the ways of reducing the challenges brought on when using these online interventions.
• We explore user centered approaches to developing online mental health interventions for students in higher education.

## 1 Introduction

A survey carried out by the World Health Organization (Auerbach et al., 2018) across 19 colleges and 8 countries revealed that approximately 31% of students experienced mental disorders (including mood disorders, anxiety disorders and substance abuse disorders). Major depressive episodes and generalized anxiety disorder were the most common mental disorders affecting the survey respondents, accounting for approximately 18% and 16% of respondents, respectively.

Despite the prevalence of mental disorders among college students, a survey carried out by the Healthy Minds project (Eisenberg et al., 2012) across 26 colleges in the United States found that only 36% of students who screened positive for a mental disorder had sought any form of treatment in the previous year. Similarly, in a much larger study by the World Health Organization across 19 colleges and 8 countries (Ebert et al., 2019), only 24.6% of college students mentioned they would seek help if faced with an emotional problem in the future.

College students face several attitudinal barriers towards seeking mental health treatment, including stigma (Eisenberg et al., 2012; Ebert et al., 2019), their perceived level of need of mental health support (Eisenberg et al., 2012), the desire to handle the problem on their own (Eisenberg et al., 2012; Ebert et al., 2019), preference for speaking to friends/family (Ebert et al., 2019), and a lack of belief that the counselors/therapists would be able to understand their situation (Eisenberg et al., 2012). In addition, they face structural barriers towards seeking mental health treatment including the cost of treatment, time, transportation and scheduling (Ebert et al., 2019).

In recent years, there has been an increase in demand for university mental health services (Auerbach et al., 2018; Gallagher, 2014; Lipson et al., 2019), leading to long waiting lists and rationing of services (Gallagher, 2014; Karwig et al., 2015). This creates a need for online mental health interventions which have been known to circumvent some of the barriers associated with face to face mental health services including stigma, cost, accessibility and time (Renton et al., 2014).

There have been a number of systematic reviews on digital mental health interventions for college students. Lattie et al. (Lattie et al., 2019) examined digital mental health interventions used among college students with a focus on the effectiveness, uptake, usability and acceptability of these interventions. Johnson et al. (Johnson and Kalkbrenner, 2017) explored mobile health interventions used to support college students’ mental health. The authors present results on the types of mobile health platforms used and the information provided to students via those platforms.

Montagni et al. (2020) carried out a review of mental-health related digital use among college students, presenting results on the aim of the interventions, barriers to use, and the advantages of digital interventions. Lastly, Organ et al. (2018) explored user design practices in illicit substance abuse interventions for college students. They presented results on user experience including user satisfaction after the intervention, needs assessment of users, and user engagement in the design of the intervention.

Although, online mental health interventions have proven effective among college students (Lattie et al., 2019), low adherence^1^ and high attrition^2^ rates are significant issues that limit the effectiveness (i.e. the ability to produce an improvement in psychological outcome variables) of online mental health interventions (Lattie et al., 2019; Becker and Torous, 2019). In a review of online mental health interventions in the general population, Borghouts et al. (2021) found that the content of the intervention, personalization and level of guidance (for example, from a human therapist or similar) were factors that affected users’ engagement with an intervention. Similarly, Torous et al. (Torous et al., 2018) carried out a review on user engagement with mental health apps. They identified factors leading to low user engagement^3^ including 1) apps were not designed with users in mind 2) apps did not solve problems users cared about 3) apps did not respect privacy 4) apps were not perceived as trustworthy 5) apps were not useful in emergency situations. These themes point towards developing online mental health interventions with a User-Centered Design (UCD) process - an approach to the development of an intervention informed by needs of the end users. The UCD process provides an opportunity to meet user's needs and expectations and improve the effectiveness of interventions (i.e. the ability to produce an improvement in the designated outcome variables) (McCurdie et al., 2012), it could also improve the engagement of end users with the technology (Lattie et al., 2019).

Consequently, our review aims to explore user-centered approaches to designing online mental health interventions specifically for college students/students in higher education/post-secondary students. In the rest of this review, we refer to this category of students as “students in higher education”.

We present results on the user-centered design practices in these interventions, design frameworks applied in the development of the intervention, engagement^4^ of students in the design process, and the usability of the interventions. We also present a qualitative synthesis of student's needs in relation to the digital mental health interventions.

We have not found any existing reviews that presented these results or studied user-centered design practices for depression, anxiety, and psychological wellbeing among students in higher education.

Therefore, in this study, we attempt to answer the following research questions:

1. What type of online interventions have been designed for depression, anxiety, and overall mental well-being for students in higher education?
2. What design methodologies are currently applied in the design process?
3. How successful are these methods in terms of user engagement and acceptance?

This review is intended to contribute to the future design and development of online mental health interventions (for depression, anxiety and overall mental wellbeing) specifically for students in higher education, while applying user centered design practices.

## 2 Methods

### 2.1 Data gathering

#### 2.1.1 Scoping review methodology

We performed our scoping review based on the framework presented by Arksey and Malley (Arksey and O’Malley, 2005). We carried out a scoping review because we wanted to give a broad overview of research published in this area. The framework includes five steps: consultation with experts; defining the research question; selecting databases; selecting studies; and charting the data. Firstly, we consulted with a librarian with expertise in mental health who provided resources that guided us in conducting this review. Following this, we defined the PICO (Population, Intervention, Control, and Outcome) and research questions for our study. In defining our research question, we prepared keywords that would guide our search in electronic databases. These keywords and research questions were refined by researchers in the field of Digital Mental Health, Psychology, and Information Technology. The search strings are included in Appendix A.

#### 2.1.2 Search strategy

The selected electronic databases for our study were also refined with input from researchers in the aforementioned fields (in Section 2.1.1). The databases we selected include Embase, ACM digital library, Web of Science, IEEE Explore, SCOPUS, EBSCO Host (including APA PyscInfo, CINAHL PLUS, APA PsycArticles, Medline), and PubMed. Preliminary searches were also carried out on Google Scholar. Further, we set up alerts in all databases, to keep track of new studies that were published while the review was ongoing. No new studies were added to the scoping review after March 15, 2021.

All databases were searched from database inception between 13 Jan 2021 and 14 Jan 2021. Our search resulted in a total of 746 articles and database alerts led to the inclusion of 9 more articles. The exclusion of duplicates led to 673 articles. We kept track of our results using the Rayyan (Ouzzani et al., 2016) systematic review software.

#### 2.1.3 Inclusion and exclusion criteria

The next stage of our review was the application of the inclusion and exclusion criteria for selecting studies. 616 articles were excluded through the review of their abstracts. Articles for which we were unsure of their inclusion were included in the Maybe section (57 articles) of the Rayyan software (Ouzzani et al., 2016). In addition, the full text of all articles in the Maybe section was studied to determine if they matched the inclusion criteria and if not they were excluded. The aforementioned screening process was conducted by the first author.

The inclusion criteria for our study are:

1. Interventions that focused on design for the improvement of mental wellbeing for students in higher education e.g. user-centered design, participatory design, etc.
2. Interventions that focus on improving mental wellbeing/depression/anxiety symptoms.
3. Interventions focused on students in higher education.
4. Intervention that are online e.g. mobile apps, web-based, etc.
5. Studies that were peer-reviewed and published in the English language. No limits were placed on the year of publication, gender, or age of the participants.

Studies not matching the aforementioned inclusion criteria were excluded. We found a number of studies (Fitzpatrick et al., 2017; Räsänen et al., 2016; Cavanagh et al., 2013) where usability was measured as part of a clinical trial, however, no user centered approaches were followed. Also, one study was excluded because it focused solely on mental health prevention (Levin et al., 2016).

No additional articles were found through hand searches in the reference lists of the included articles. The first author performed the handsearches, while both authors discussed the articles for which the first author was unsure of their decision.

Finally, 24 articles were included in the scoping review as of 18 February 2021. One further article was excluded because it was a protocol study for which no corresponding design paper was found.

**Fig. 1:**
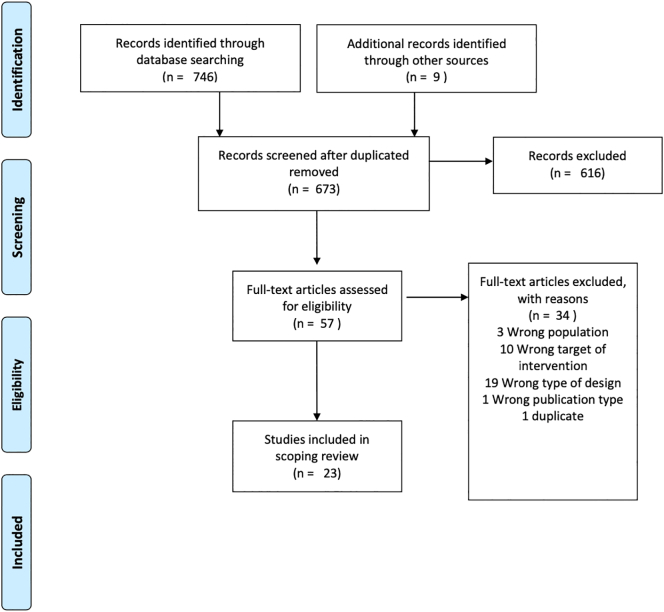
PRISMA flow diagram.

Fig. 1 shows the PRISMA diagram for our scoping review.

#### 2.1.4 Data extraction and analysis

##### 2.1.4.1 Data items

To answer the research questions, we charted information from the included studies and grouped them into the following categories. The data characteristics for all included studies are presented in Table 1.

**Table 1:** Characteristics of included studies.

Author (Year)	Name/Target/Type/Development Stage/Duration of Intervention	UCD methods	Design Process/Framework	Measure of Engagement
Hookham et al. (2016)	*Name*: Shadow *Target*: Comorbidity of depression and alcohol use disorders *Type*: Desktop program *Stage*: Developed, tested in a pilot *Duration*: N/A	• User survey • Think aloud	*Process*: None described *Framework*: None listed	• System Usability Scale • Perceived Usefulness and Ease of Life survey • Game Engagement Survey
Costa et al. (2016)	*Name*: Emotioncheck *Target*: Mental health and anxiety *Type*: Mobile and wearable sensing *Stage*: Developed, tested in a pilot *Duration*: One-day period	• User survey	*Process*: None described *Framework*: None listed	None/Financial incentive given
Báldy et al. (2020)	*Name*: Unnamed *Target*: Social Anxiety Disorder (SAD) *Type*: Desktop program *Stage*: Developed, tested in a pilot *Duration*: 2 weeks	• Literature review • Interview • User survey • Contextual inquiry	*Process*: None described *Framework*: None listed	• User Engagement Scale- Short Form • Narrative Engagement Scale • Recall Questionnaire • Interviews
Currie et al. (2010)	*Name*: Feeling Better *Target*: Depression, anxiety, and stress *Type*: Web-based platform *Stage*: Developed, tested in a pilot *Duration*: One-day period	• Interview • Usability testing	*Process*: Iterative usability testing cycles with students and counseling staff *Framework*: Usability testing framework (Kushniruk, 2002)	None/Financial incentive given
Rodgers et al. (2019)	*Name*: MoodCloud *Target*: Overall wellbeing *Type*: Mobile app and ambient display *Stage*: Developed, tested in a pilot *Duration*: 1 week	• Interview • Log data review	*Process*: None described *Framework*: None listed	None
Kim et al. (2020)	*Name*: Gloomy *Target*: Mental health and stigma reduction *Type*: web-based/mobile platform *Stage*: Developed, tested in a pilot *Duration*: 3 weeks	• Contextual inquiry • Interview • Log data review	*Process*: It was developed using Cognitive Behavior Therapy (CBT) principles and reviewed by psychologists. Also, the personal story content from the social bot was developed with the help of four clinical psychologists who work at the university counseling center. *Framework*: None listed	None/Financial incentive given
Huang et al. (2015)	*Name*: Emotionmap *Target*: Emotion regulation *Type*: Mobile app *Stage*: Developed, tested in a pilot *Duration*: 4 weeks	• Contextual inquiry • Interview • Literature review • Log data review	*Process*: The design of the app was based on emotion-regulation theories. Also, staff from the university counseling center and a professor in clinic-health psychology were consulted in the development process. *Framework*: None listed	None/Financial incentive given
Ananthabhotla et al. (2017)	*Name*: MIT community challenge *Target*: Overall wellbeing *Type*: Web-based platform *Stage*: Developed, tested in a pilot *Duration*: 12 days	• Interview • Log data review	*Process*: None described *Framework*: None listed	None
Khan and Pea (2017)	*Name*: Unnamed *Target*: Depression *Type*: Desktop program *Stage*: Developed, tested in a pilot *Duration*: One-day period	• User survey • Log data review	*Process*: Content based on neuro-physiological training tasks proven to help improve cognitive control in people with depression *Framework*: None listed	• Time spent on the app/Financial incentive given
Doherty et al. (2012)	*Name*: Silvercloud (Mindbalance) *Target*: Depression *Type*: Web-based platform *tage*: developed, evaluated in a clinical trial *Duration*: 8 weeks	• User survey • Interview • Log data review	*Process*: Content developed in collaboration with clinical psychologists, psychiatrists and psychotherapists, design workshops were carried out with primary care, specialized referral services, patients, youth panels from NGOs. Also, a pilot study was carried out before this clinical study *Framework*: None listed	• Time spent in app • Participation in last week of intervention
Wiljer et al. (2016)	*Name*: ThoughtSpot Protocol *Target*: Mental health and wellbeing *Type*: N/A *Stage*: Protocol *Duration*: N/A	N/A	*Process*: Design is based on social-cognitive theory and theory of help-seeking. *Framework*: Participatory Design of Evidence-based Online Youth Mental Health Promotion, Prevention, Early Intervention, and Treatment (Hagen et al., 2012)	N/A
VanHeerwaarden et al. (2018)	*Name*: ThoughtSpot (participatory design) *Target*: Mental health and wellbeing *Type*: Mobile and web-based platform *Stage*: Developed, tested in a pilot *Duration*: N/A	• User survey • Co-design workshop	*Process*: Student led workshops including semi-structured focus groups, questionnaires, personas, journey mapping and world café *Framework*: Participatory Design of Evidence-based Online Youth Mental Health Promotion, Prevention, Early Intervention, and Treatment (Hagen et al., 2012)	N/A
Wiljer et al. (2020)	*Name*: ThoughtSpot (randomized control trial) *Target*: Mental health and wellbeing *Type*: Mobile and web-based platform *Stage*: Developed, evaluated in a clinical trial *Duration*: 6 months	• User survey • Log data review	*Process*: Student led workshops led to the improvement of the original ThoughtSpot which is the version presented in this study *Framework*: Participatory Design of Evidence-based Online Youth Mental Health Promotion, Prevention, Early Intervention, and Treatment (Hagen et al., 2012)	• Continuance in study • Continued use of platform • Financial incentive given
Hickie et al. (2019)	*Name*: FitUniLife To Thrive *Target*: Overall wellbeing *Type*: Mobile and web-based platform *Stage*: Prototype *Duration*: N/A	• Co-design workshop	*Process*: Co-design workshops, feasibility testing, user testing and implementation. Co-design involved young people, supportive others, health professionals and service providers. *Framework*: Participatory Design of Evidence-based Online Youth Mental Health Promotion, Prevention, Early Intervention, and Treatment (Hagen et al., 2012)	N/A
Coughlan et al. (2019)	*Name*: Our Journey *Target*: Mental health and wellbeing *Type*: Web-based platform *Stage*: Developed, not tested in a pilot *Duration*: N/A	• Co-design workshop	*Process*: Participatory design with students, student union representatives, and staff (in student support roles, and those supporting disabled students) *Framework*: None listed	N/A
Meng et al. (2018)	*Name*: ISee *Target*: Depression *Type*: Mobile sensing and a Mobile app *Stage*: Prototype *Duration*: N/A	• Co-design workshop	*Process*: Participatory design workshop with clinicians and students *Framework*: None listed N/A	
Gulliver et al. (2015)	*Name*: UniVirtual Clinic (understanding privacy needs) *Target*: Mental health *Type*: Mobile app *Stage*: Prototype *Duration*: N/A	• Focus group • Prototype testing	*Process*: Focus groups with students and iterative prototype testing sessions with students *Framework*: None listed	N/A
Farrer et al., (2015)	*Name*: UniVirtual Clinic (end-user needs) *Target*: Mental health *Type*: Mobile app *Stage*: Prototype *Duration*: N/A	• Focus group	*Process*: Focus groups with students *Framework*: None listed	N/A
Farrer et al., (2020)	*Name*: UniVirtual Clinic *Target*: Mental health *Type*: Web-based platform *Stage*: Developed, not tested in a pilot *Duration*: N/A	• Focus group • Literature review • User survey • Prototype testing	*Process*: Participatory design with young people, university stakeholders and other service providers *Framework*: None listed	N/A
Morr et al. (2020)	*Name*: Unnamed *Target*: Mental health *Type*: Online intervention (type not mentioned by authors) *Stage*: Developed, not tested in a pilot *Duration*: N/A	• Focus group	*Process*: Focus groups with students *Framework*: None listed	N/A
Kajitani et al. (2020)	*Name*: Mental App *Target*: Mental health *Type*: Mobile app *Stage*: Developed, tested in a pilot *Duration*: 2 weeks	• User survey • Log data review	*Process*: Questionnaires with university students. *Framework*: None listed	• Log data • Usability survey
Yoo and Choudhury (2019)	*Name*: Unnamed *Target*: Mental health *Type*: Web and desktop platform *Stage*: Prototype *Duration*: N/A	• Interview • Think aloud • Prototype testing	*Process*: Interviews and prototype sessions with campus administrators and campus clinicians *Framework*: None listed	N/A
Papadatou-Pastou et al. (2019)	*Name*: MePlusMe *Target*: Mental health, wellbeing and study skills *Type*: Desktop (offline) with intent to convert to an app *Stage*: Developed, tested in a pilot *Duration*: 8 weeks	• User survey • Interview	*Process*: Survey with students, interviews with staff at counseling services (Goozee et al., 2018). Proof of concept with students and university executives (Touloumakos et al., 2016). *Framework*: None listed	• Number of participants completing the intervention

###### 2.1.4.1.1 Year of publication

The year in which the study was published.

###### 2.1.4.1.2 Name of intervention

This refers to the name given to the intervention. If the intervention had no name, it was listed as “Unnamed”.

###### 2.1.4.1.3 Target of intervention

The aspect (s) of mental health that the intervention is focused on.

###### 2.1.4.1.4 Type of online intervention

The type of online intervention refers to the medium through which the intervention was delivered, for example, via mobile or web-based applications.

###### 2.1.4.1.5 Development stage of the intervention

This refers to the development stage of the intervention in the published study. The stages include protocol, prototype, developed app/web-based program, developed and tested in a pilot, developed and evaluated in a Randomized Controlled Trial (RCT).

###### 2.1.4.1.6 Duration of the intervention

This refers to the length of time in which participants’ used the intervention in a research setting.

###### 2.1.4.1.7 User-Centered Design (UCD) methods

This refers to user-centered design practices applied in the development of the intervention. This includes user surveys, interviews, focus groups, personas (a realistic representation of a segment of end-users (Usability.gov, Oct 2013)), log data reviews, prototyping, usability testing etc. More information on user-centered design methods can be found in (Usability.gov, n.d.).

###### 2.1.4.1.8 Design process

This refers to the design process of the intervention.

###### 2.1.4.1.9 Design framework

This refers to frameworks used as guidelines during the design and development of the intervention.

###### 2.1.4.1.10 Measure of engagement

This refers to tools used in measuring level of user engagement (regardless of authors’ definition of engagement). In addition, we include information about whether financial incentives were given to participants in the study as it could affect participants’ engagement in the intervention (Organ et al., 2018).

## 3 Results

Our scoping review yielded a total of 23 articles, some of which belong to the same intervention. Since one of our research questions was to understand the design methodologies applied in the interventions, it made sense to look at the interventions holistically, if possible. This applied to the intervention called ThoughtSpot, for which we included the protocol (Wiljer et al., 2016), the participatory design process, (VanHeerwaarden et al., 2018) and the randomized controlled trial (Wiljer et al., 2020). Similarly, for the intervention UniVirtualClinic, we included the participatory design process (Gulliver et al., 2015; Farrer et al., 2015) and development of the intervention in our review (Farrer et al., 2020). Consequently, our scoping review yielded a total of 19 unique interventions. Upon charting our data, we present the following results.

### 3.1 Year of publication

All included studies were published between the years 2010 and 2020, as shown in Table 2. (Currie et al., 2010) was published in 2010, (Doherty et al., 2012) was published in 2012, (Huang et al., 2015; Gulliver et al., 2015; Farrer et al., 2015) were published in 2015, (Hookham et al., 2016; Costa et al., 2016; Wiljer et al., 2016) were published in 2016, (Ananthabhotla et al., 2017; Khan and Pea, 2017) were 2017, (VanHeerwaarden et al., 2018; Meng et al., 2018) were published in 2018, (Rodgers et al., 2019; Hickie et al., 2019; Coughlan et al., 2019; Yoo and Choudhury, 2019; Papadatou-Pastou et al., 2019) were published in 2019, and (Báldy et al., 2020; Kim et al., 2020; Wiljer et al., 2020; Farrer et al., 2020; Morr et al., 2020; Kajitani et al., 2020) were published in 2020.

**Table 2:** Studies and years of publication.

Year of Publication	Name of Intervention (s)
2010	FeelingBetter (Currie et al., 2010)
2012	SilverCloud – MindBalance (Doherty et al., 2012)
2015	EmotionMap (Huang et al., 2015), UniVirtualClinic (Gulliver et al., 2015; Farrer et al., 2015).
2016	Shadow (Hookham et al., 2016), EmotionCheck (Costa et al., 2016), ThoughtSpot (Wiljer et al., 2016).
2017	MIT community challenge (Ananthabhotla et al., 2017), unnamed (Khan and Pea, 2017).
2018	ThoughtSpot (VanHeerwaarden et al., 2018), ISee (Meng et al., 2018).
2019	MoodCloud (Rodgers et al., 2019), FitUniLife (Hickie et al., 2019), Our Journey (Coughlan et al., 2019), unnamed (Yoo and Choudhury, 2019), MePlusMe (Papadatou-Pastou et al., 2019).
2020	unnamed (Báldy et al., 2020), Gloomy (Kim et al., 2020), ThoughtSpot (Wiljer et al., 2020), UniVirtualClinic (Farrer et al., 2020), unnamed (Morr et al., 2020), unnamed (Kajitani et al., 2020).

### 3.2 Age of participants

A majority of the studies included participants between 18 and 30 years (Hookham et al., 2016; Costa et al., 2016; Currie et al., 2010; Rodgers et al., 2019; Kim et al., 2020; Khan and Pea, 2017; VanHeerwaarden et al., 2018; Hickie et al., 2019; Meng et al., 2018; Gulliver et al., 2015; Farrer et al., 2015; Farrer et al., 2020). Two studies included a mean age of participants without mentioning the age range of participants. Báldy et al. (2020) noted that the mean age of their participants was 24.2 while for Papadatou-Pastou et al. (2019) the mean age of participants was 31.3.

Further, (Wiljer et al., 2016; Wiljer et al., 2020) allowed for slightly younger participants with a range of 16-30 and 17-30 respectively. On the other hand, Morr et al. (2020) allowed for much older participants with a range of 18-50. This is significant because a majority of studies on digital mental health interventions in higher education inadvertently exclude older students. A number of the studies (Huang et al., 2015; Ananthabhotla et al., 2017; Doherty et al., 2012; Coughlan et al., 2019; Kajitani et al., 2020; Yoo and Choudhury, 2019) included no information on the ages of their participants.

### 3.3 Research question 1

What type of online interventions have been designed for the improvement of depression symptoms, anxiety symptoms, and overall mental well-being for students in higher education? We answer this research question under three headings - target of the intervention i.e. the area of mental health/wellbeing, the medium of delivering the intervention, and the development stage of the intervention.

#### 3.3.1 Target of intervention

The included articles targeted a variety of areas in mental health and wellbeing, including emotion regulation, an aspect of treatment in depression (Huang et al., 2015). Other targeted areas include depression (Khan and Pea, 2017; Doherty et al., 2012; Meng et al., 2018), mental health (Gulliver et al., 2015; Farrer et al., 2015; Farrer et al., 2020; Morr et al., 2020; Kajitani et al., 2020; Yoo and Choudhury, 2019), mental health and wellbeing (Wiljer et al., 2016; VanHeerwaarden et al., 2018; Wiljer et al., 2020; Coughlan et al., 2019), depression and alcohol use disorders (Hookham et al., 2016), depression, stress and anxiety (Currie et al., 2010), mental health and anxiety (Costa et al., 2016), overall wellbeing (Rodgers et al., 2019; Ananthabhotla et al., 2017; Hickie et al., 2019), mental health, wellbeing and study skills (Papadatou-Pastou et al., 2019), mental health and stigma reduction (Kim et al., 2020) and lastly, social anxiety disorder (Báldy et al., 2020). As can be seen from the targeted areas of various studies, many studies consider multiple areas of mental health. We have only included studies that targeted at least one area of our interest i.e. depression, anxiety or overall mental wellbeing.

#### 3.3.2 Medium of delivering intervention

The interventions were delivered online via mobile apps (Rodgers et al., 2019; Kim et al., 2020; Huang et al., 2015; Wiljer et al., 2020; Hickie et al., 2019; Meng et al., 2018; Kajitani et al., 2020), desktop apps (Hookham et al., 2016; Báldy et al., 2020; Khan and Pea, 2017; Yoo and Choudhury, 2019), web-based platforms (Currie et al., 2010; Kim et al., 2020; Ananthabhotla et al., 2017; Doherty et al., 2012; Wiljer et al., 2020; Hickie et al., 2019; Coughlan et al., 2019; Farrer et al., 2020; Yoo and Choudhury, 2019), wearable devices (Costa et al., 2016), and a tangible ambient display (Rodgers et al., 2019). In the case of MePlusMe (Papadatou-Pastou et al., 2019), based on feedback from participants, the authors intended to make the offline desktop program an online intervention, therefore, it was included in our review. It should be noted that some interventions were delivered via multiple mediums, for instance, Gloomy (Kim et al., 2020) had a mobile and a web-based app. On the other hand, the online mindfulness virtual community (Morr et al., 2020) was in the prototype stage of development, and the intended medium was not specified by the authors, although, they mention it would be developed as an online intervention.

#### 3.3.3 Development stage of intervention

The interventions of included studies were at different stages of development. Some of the interventions were study protocols (Wiljer et al., 2016); protocols were only included if they applied user-centered practices or if part of the intended study was already completed and published. Some of the studies involved the design and improvement of prototypes (Hickie et al., 2019; Meng et al., 2018; Gulliver et al., 2015; Farrer et al., 2015; Yoo and Choudhury, 2019), in others, the interventions (incl. mobile/web/desktop apps) were developed and being tested in a pilot study (Hookham et al., 2016; Costa et al., 2016; Báldy et al., 2020; Currie et al., 2010; Rodgers et al., 2019; Kim et al., 2020; Huang et al., 2015; Ananthabhotla et al., 2017; Khan and Pea, 2017; VanHeerwaarden et al., 2018; Kajitani et al., 2020; Papadatou-Pastou et al., 2019). In other studies, although the intervention had been developed, it was not yet tested in a pilot study (Coughlan et al., 2019; Farrer et al., 2020; Morr et al., 2020). Finally, in MindBalance (Doherty et al., 2012) and Thoughtspot (Wiljer et al., 2020), the intervention had undergone a clinical trial. Table 3 shows the interventions and the stages of development. It should be noted that some of the interventions are mentioned more than once because more than one stage of development was included in the review.

**Table 3:** Development stage of intervention (s).

Development Stage of Intervention	Name/Pseudonym of Intervention (s)
Developed, evaluated in a clinical trial	Silvercloud-mindbalance (Doherty et al., 2012), ThoughtSpot-clinical trial (Wiljer et al., 2020).
Developed, tested in a pilot	Shadow (Hookham et al., 2016), EmotionCheck (Costa et al., 2016), unnamed (Báldy et al., 2020), FeelingBetter (Currie et al., 2010), MoodCloud (Rodgers et al., 2019), Gloomy (Kim et al., 2020), EmotionMap (Huang et al., 2015), MitCommunityChallenge (Ananthabhotla et al., 2017), BeatTheBlues (Khan and Pea, 2017), ThoughtSpot Partcipatory design (VanHeerwaarden et al., 2018), unnamed (Kajitani et al., 2020), MePlusMe (Papadatou-Pastou et al., 2019).
Developed, not tested in a pilot	UniVirtualClinic (Farrer et al., 2020), Our Journey (Coughlan et al., 2019), Mindfulness Virtual Community (Morr et al., 2020).
Prototype	ISee (Meng et al., 2018), UniVirtualClinic-Privacy (Gulliver et al., 2015), FitUniLife (Hickie et al., 2019), UniVirtualClinic-needs (Farrer et al., 2015), unnamed (Yoo and Choudhury, 2019).
Protocol	ThoughtSpot Protocol (Wiljer et al., 2016).

### 3.4 Research question 2

What design methodologies are currently applied in the design process? In this question, we focus on what kind of design frameworks were applied, the engagement of students/other stakeholders in the design of the interventions and the methods of engaging stakeholders.

#### 3.4.1 Inclusion of stakeholders

In a review of frameworks for the development of eHealth interventions, (van Gemert-Pijnen et al., 2011) note that the inclusion of stakeholders is pertinent to “reflect the values, drivers and goals of an eHealth intervention”. This could be implemented via design workshops, persona building, surveys, focus groups, interviews, etc. Prior to designing/developing an intervention, the inclusion of stakeholders involves understanding the needs of the users and the environment where the intervention will be delivered - *a contextual inquiry*.

Eleven of the included interventions (Báldy et al., 2020; Kim et al., 2020; Huang et al., 2015; Doherty et al., 2012; Wiljer et al., 2020; Hickie et al., 2019; Coughlan et al., 2019; Farrer et al., 2020; Morr et al., 2020; Yoo and Choudhury, 2019; Papadatou-Pastou et al., 2019) mention an initial contextual inquiry with stakeholders (including users) to understand the needs of the users and the environment in which the intervention will be deployed.

In addition, in (Báldy et al., 2020; Kim et al., 2020; Huang et al., 2015; Hickie et al., 2019; Coughlan et al., 2019; Farrer et al., 2020; Kajitani et al., 2020; Yoo and Choudhury, 2019), stakeholders were involved in the contextual inquiry (requirement gathering process) and in the current stage of the intervention - in building the prototypes (Hickie et al., 2019; Coughlan et al., 2019; Farrer et al., 2020; Yoo and Choudhury, 2019) or the pilot study (Báldy et al., 2020; Kim et al., 2020; Huang et al., 2015; Kajitani et al., 2020).

Further, in ThoughtSpot (Wiljer et al., 2020), stakeholders were involved in the contextual inquiry, pilot study, and randomized controlled trial. Similarly, in MePlusMe (Papadatou-Pastou et al., 2019), stakeholder input was present in the contextual inquiry, prototype building and the pilot study (which was the current stage of development of the intervention). Lastly, in SilverCloud-MindBalance (Doherty et al., 2012), stakeholders were involved in all stages of the intervention including the contextual inquiry, prototype building, the pilot study, and in the clinical trial.

#### 3.4.2 Type of stakeholder

The type of stakeholders included in contextual inquiries differed across the studies, including students (Wiljer et al., 2020; Hickie et al., 2019; Coughlan et al., 2019; Farrer et al., 2020; Morr et al., 2020; Papadatou-Pastou et al., 2019), youth in the general population (Doherty et al., 2012; Hickie et al., 2019), student union representatives (Coughlan et al., 2019), university staff in various roles (Huang et al., 2015; Hickie et al., 2019; Coughlan et al., 2019; Farrer et al., 2020; Yoo and Choudhury, 2019), and counseling staff (Báldy et al., 2020; Kim et al., 2020; Huang et al., 2015; Doherty et al., 2012; Farrer et al., 2020; Yoo and Choudhury, 2019; Papadatou-Pastou et al., 2019). A majority of the studies included different types of stakeholders in their contextual inquiries.

#### 3.4.3 Method of stakeholder engagement

All studies involved some form of stakeholder input using various approaches. Firstly, usability questionnaires (Hookham et al., 2016; Costa et al., 2016; Báldy et al., 2020; Khan and Pea, 2017; Doherty et al., 2012; Wiljer et al., 2020; Farrer et al., 2020; Kajitani et al., 2020; Papadatou-Pastou et al., 2019) and interviews (Báldy et al., 2020; Currie et al., 2010; Rodgers et al., 2019; Kim et al., 2020; Huang et al., 2015; Ananthabhotla et al., 2017; Doherty et al., 2012; Wiljer et al., 2020; Meng et al., 2018; Yoo and Choudhury, 2019) were used to assess participants’ experiences following their use of the technological intervention. In addition, startle reflex modulation (Hookham et al., 2016), think aloud (Hookham et al., 2016; Currie et al., 2010; Yoo and Choudhury, 2019), and usage logs (Rodgers et al., 2019; Kim et al., 2020; Huang et al., 2015; Ananthabhotla et al., 2017; Doherty et al., 2012; Kajitani et al., 2020), were used to assess the participants’ experiences as they used the intervention.

Also, some studies applied psychological questionnaires (Kim et al., 2020; Khan and Pea, 2017; Doherty et al., 2012; Wiljer et al., 2020; Kajitani et al., 2020; Papadatou-Pastou et al., 2019), to evaluate the impact of the intervention on the users’ mental well-being.

Furthermore, recall questionnaires (Báldy et al., 2020) were used to test the ability of the user to remember what they learned while using the intervention. Lastly, prototype testing sessions (Gulliver et al., 2015; Yoo and Choudhury, 2019), focus groups (Farrer et al., 2020; Morr et al., 2020) and co-design workshops (VanHeerwaarden et al., 2018; Hickie et al., 2019; Coughlan et al., 2019) were used to actively include stakeholder input in the design process.

#### 3.4.4 Design framework

Only three of the included interventions mention applying a design framework in the development of their online intervention. ThoughtSpot (Wiljer et al., 2020) and FitUniLife (Hickie et al., 2019) apply the guidelines on *Participatory Design of Evidence-based Online Youth Mental Health Promotion, Prevention, Early Intervention, and Treatment* by the Young and Well Cooperative Research Centre (Hagen et al., 2012). This framework advocates for the development of interventions using input from young people and scholarly evidence. The framework is guided by three main principles 1) Young people should actively participate throughout the design process, from problem setting to problem solving 2) Young people should participate in idea generation and provide feedback on existing design concepts 3) Proposed interventions should be continuously evaluated from the perspective of the end-users on whether they are relevant, meaningful and engaging. In addition, the potential for causing harm and the impact on mental health and wellbeing should be taken into account (Hagen et al., 2012).

In ThoughtSpot, researchers operationalize this framework through a review of literature on social-cognitive theory and theory on help-seeking behavior. Young people were involved from the beginning of the research project, contributing to decisions on the project name, the logo of ThoughtSpot, and product design. In addition, five co-design workshops (with 41 young people) were carried out to improve ThoughtSpot exploring: usage of eHealth apps and user experience of ThoughtSpot for new users of the program; usage of eHealth apps and user experience of ThoughtSpot for experienced users of the program; whether ThoughtSpot met the needs of the designed user personas (a realistic representation of a segment of end-users (Usability.gov, 2013)); the interaction of new users with the program; what new features could be added to the program, the needs currently being addressed by the program and how to keep users coming back. Lastly, a usability questionnaire was used to assess the user experience following the RCT. Similarly, in FitUniLife (Hickie et al., 2019), the researchers explored scholarly evidence on core features for inclusion in an online health and wellbeing system. In addition, they carried out three co-design workshops (with 15 staff and 31 students) exploring: internet and hardware use and prototype building; specific user needs to improve the prototype; and addressing whether the prototype will meet the health and wellbeing needs of end users.

Further, ThoughtSpot (Wiljer et al., 2020) applies principles from *Participatory Action Research (PAR)* (Baum et al., 2006) which focuses on the active participation of end users in the development of an intervention, where the “researched become researchers”. It involves a reflexive cycle of data collection and analysis that informs action (action from end-users to improve their own health). PAR was evidenced in ThoughtSpot through the development of the initial version of the program by 65 university students, involvement of students in project management and involvement of students in the organization of the co-design workshops.

Finally, the intervention FeelingBetter (Currie et al., 2010) applies Andre Kushniruk's (Kushniruk, 2002) guidelines on usability engineering for Health Information Systems. This guideline states that usability testing (an evaluation of a system through the analysis of end-users interacting with the system) should be applied in the development of an health information system. In FeelingBetter (Currie et al., 2010), researchers carried out three cycles of usability testing with staff and students in order to point out usability problems in their program. Participants were asked to “think aloud” while interacting with the system and feedback from each cycle was used to redesign the program.

### 3.5 Research question 3

How successful are these methods in terms of user engagement and acceptance? To answer this question we discuss the definitions of engagement across the included studies, we examine attrition rates as a measure of engagement, we review user acceptance themes in the studies, and lastly, we review the definition of success in these online mental health interventions.

#### 3.5.1 Defining engagement

We consider the definitions of engagement in the included interventions, focusing on interventions that were developed and tested in a pilot study, and/or evaluated in a clinical trial. In Shadow (Hookham et al., 2016), the authors designed a gamified CBT application for depression and alcohol use disorders. They define engagement as a progression of the following concepts: immersion - “experience of becoming absorbed in game play while having an awareness of one's surroundings” (Hookham et al., 2016; Brockmyer et al., 2009); presence - “experience of being part of a virtual environment” (Hookham et al., 2016; Brockmyer et al., 2009); flow - “experience of focusing mainly on the task at hand with limited awareness of one's surroundings” (Hookham et al., 2016); and absorption - “experience of total engagement in game play” (Hookham et al., 2016). In Shadow (Hookham et al., 2016), engagement is measured using an adapted version of the Game Engagement Survey (Brockmyer et al., 2009) in which immersion, presence, flow and absorption are measured.

Similarly, Baldy et al. (Báldy et al., 2020) designed a serious game to raise awareness on CBT skills associated with the treatment of Social Anxiety Disorder. They consider two forms of engagement in their study - user engagement and narrative engagement. According to O’Brien (2016), user engagement is defined as “a quality of user experience characterized by the depth of an actor's cognitive, temporal, affective and behavioral investment when interacting with a digital system”. In addition, narrative engagement is defined by a combination of constructs including: sympathy - “feeling sorry for the characters in the game” (Busselle and Bilandzic, 2009); empathy - “understanding what the characters are experiencing” (Busselle and Bilandzic, 2009); cognitive perspective taking - “understanding why the characters in the game felt the way they felt” (Busselle and Bilandzic, 2009); narrative presence - “being closer to the story world than the real world” (Busselle and Bilandzic, 2009) and flow - “being completely immersed in the story world” (Busselle and Bilandzic, 2009). In (Báldy et al., 2020), engagement is measured using the User Engagement Scale in short form (O’Brien et al., 2018), the Narrative Engagement Scale (Busselle and Bilandzic, 2009) and an interview.

On the other hand, (Kim et al., 2020; Doherty et al., 2012; Papadatou-Pastou et al., 2019) define engagement as the ability to retain participants in the last week of their pilot study/clinical trial. This is a common definition of engagement in CBT-related applications. In Gloomy (Kim et al., 2020) and Silvercloud- MindBalance (Doherty et al., 2012), participation in the last week of the intervention was noted via log data review, while in MePlusMe (Papadatou-Pastou et al., 2019), it was noted through participation in a usability survey at the end of the study.

Further, in ThoughtSpot (Wiljer et al., 2020), participants were expected to rate, review or add mental health and wellbeing spots to a mapping app. Therefore, the authors define engagement to be participants’ active participation in the app, a similar definition applies to MIT Community Challenge (Ananthabhotla et al., 2017) and Mental App (Kajitani et al., 2020). Log data review was used to monitor active participation across all studies (Ananthabhotla et al., 2017; Wiljer et al., 2020; Kajitani et al., 2020). Similarly, in EmotionMap (Huang et al., 2015) and BeatTheBlues (Khan and Pea, 2017), log data review was used to calculate the time spent by participants in the app, which was the authors’ definition of engagement.

#### 3.5.2 Attrition rates

User engagement is usually quantified in the context of dropout rates/retention rates. In online mental health interventions, this measure is important as lack of adherence could undermine the effectiveness of an intervention (Lattie et al., Jul 2019; Becker and Torous, 2019). Some of the included interventions were carried out over a one-day period (Hookham et al., 2016; Costa et al., 2016; Currie et al., 2010; Khan and Pea, 2017), therefore, dropout rates are not mentioned. In certain interventions with longer duration, financial incentives were provided, making it difficult to ascertain the levels of user engagement with the intervention (Kim et al., 2020; Huang et al., 2015; Wiljer et al., 2020).

Other studies do not include any information on drop-out rates (Báldy et al., 2020; Rodgers et al., 2019; Ananthabhotla et al., 2017). This may be a result of the studies taking place over a period of two weeks or less.

Table 4 shows the studies that include information on drop-out rates, including, SilverCloud-MindBalance (Doherty et al., 2012), MePlusMe (Papadatou-Pastou et al., 2019) and Mental App (Kajitani et al., 2020). Dropout rates are defined as participation in the last week of the intervention (Doherty et al., 2012; Papadatou-Pastou et al., 2019) and as the completion of the intervention (Kajitani et al., 2020).

**Table 4:** Drop-out rates across studies.

Name of Intervention	Duration of Intervention	Drop-out Rate (in %)
SilverCloud- MindBalance (Doherty et al., 2012)	8 weeks	36
Mental App (Kajitani et al., 2020)	2 weeks	20
MePlusMe (Papadatou-Pastou et al., 2019)	8 weeks	53.85

We exclude Mental App (Kajitani et al., 2020) from further analysis on engagement as we consider the duration of the intervention too small to indicate a drop-out rate. Following this, in an attempt to understand what characteristics could contribute to different rates in drop-out, we discuss the differences between the interventions MindBalance (Doherty et al., 2012) and MePlusMe (Papadatou-Pastou et al., 2019), as shown in Table 5.

**Table 5:** Differences between Silvercloud and MePlusMe.

	Silvercloud-MindBalance (Doherty et al., 2012)	MePlusMe (Papadatou-Pastou et al., 2019)
Type of Intervention	Web-based	Desktop-offline
Access to professionals	Yes	No
End user engagement	Contextual inquiry, prototype, pilot and clinical trial.	Contextual inquiry, prototype, pilot.
Stakeholder engagement	Contextual inquiry, prototype, pilot and clinical trial.	Contextual inquiry, prototype.
Final stage of development	Clinical trial	Pilot
Peer engagement	Yes	No
Number of participants in intervention	45	13

The inclusion of stakeholders (including end-users) was a significant aspect in the development of MindBalance and MePlusMe. On the contrary, the final stage in the development of MindBalance was a clinical trial as opposed to the final stage in the development of MePlusMe which was a pilot study. We believe that the extended stage of testing for MindBalance and inclusion of stakeholders (including users) throughout development could account for a lower dropout rate. As Torous et al. (2018) found, a lack of user-centered design was a contributing factor to low user engagement in mental health apps.

Also, participants in the pilot study for MePlusMe wanted the intervention to become an app-based program as opposed to an offline desktop program. The format of this intervention is not as convenient and flexible as an online intervention, this could also account for the difference in attrition rates.

In addition, Sharry et al. (2013) found that self-guided online mental health interventions had higher rates of attrition than those that included human input. This is another important difference between the two interventions as MindBalance includes access to professionals while MePlusMe does not.

Further, as can be seen in Table 6 on User Acceptance Themes, peer engagement is presented as a preference among participants in this population. This is another characteristic that could contribute to the differing rates of dropout as MindBalance includes peer engagement while MePlusMe does not.

**Table 6:** User acceptance themes - what students want?

Theme	Theme Found in	Participants’ Comment Example
Convenience	(Doherty et al., 2012; Farrer et al., 2020; Morr et al., 2020)	“I would imagine that for me an online community takes a lot of excuses I could have, like I don't want to go, or I can't fit it into my schedule, I can always go online” (Morr et al., 2020)
Add personalization	(Huang et al., 2015; Hickie et al., 2019; Meng et al., 2018; Farrer et al., 2020; Morr et al., 2020)	“My roommate and I have different style of meditation. Like, for me, I prefer like a guided meditation that talks about sensation, whereas she prefers more like, a visualization, like imagine yourself on a beach, kind of thing, so having those options will be good.” (Morr et al., 2020)
Improve interface/presentation	(Báldy et al., 2020; Currie et al., 2010; Farrer et al., 5 2020; Morr et al., 2020; Shi et al., 2021)	“Improve the animation quality. Change the camera shaking” (Báldy et al., 2020)
Change format of intervention	(Báldy et al., 2020; Papadatou-Pastou et al., 2019)	“Make it a Virtual Reality game” (Báldy et al., 2020)
Change intervention	(Huang et al., 2015; Ananthabhotla et al., 2017; Doherty et al., 2012)	“I don't like to think back. I only want to look at the present. I don't see the report has value to me” (Huang et al., 2015)
Improve content	(Kim et al., 2020; Huang et al., 2015; Ananthabhotla et al., 2017; Hickie et al., 2019; Meng et al., 2018; Farrer et al., 2020; Shi et al., 2021)	“I tend to eat more when I'm more depressed, more of a comfort food. Visualization will be helpful so that I can see really highs or lows” (Meng et al., 2018)
Ensure anonymity/privacy/safety	(Doherty et al., 2012; Meng et al., 2018; Gulliver et al., 2015; Farrer et al., 2020; Morr et al., 2020)	“I think it's important to have your username account but also a guest account so if you don't want to do something which someone else can see, you can use the private guest account, which won't record your information.” (Gulliver et al., 2015)
Add peer engagement	(Huang et al., 2015; Doherty et al., 2012; Farrer et al., 2020; Morr et al., 2020)	“I'd like to have more people using the app. I only have a few friends using it because of this I don't feel like posting many emotions, it feels like no one cares for you” (Huang et al., 2015)
Include access to professionals	(Meng et al., 2018; Farrer et al., 2020; Morr et al., 2020; Papadatou-Pastou et al., 2019)	“It might be easier for me to go through the tracked data with my doctor, so he can tell me what to do from there” (Meng et al., 2018)
Make it more engaging	(Báldy et al., 2020; Currie et al., 2010; Kim et al., 2020; Coughlan et al., 2019; Farrer et al., 2020; Morr et al., 2020; Papadatou-Pastou et al., 2019)	“Make the game more challenging” (Báldy et al., 2020)

Although the stated differences between the interventions could explain the different rates of attrition, there could be several unseen factors at play.

#### 3.5.3 User acceptance

In looking at user acceptance, we focus on the user experience of the intervention. As mentioned earlier, different studies applied different methods in understanding user experience including questionnaires, interviews, focus groups, etc. It should be noted that user acceptance here encompasses usability in general i.e. user-friendliness, ease of navigation, clarity of content, and acceptability (i.e., users like the app/program).

The quantitative aspects of usability questionnaires could be easily combined, however, they are not derived from the same sources, and in most cases, the studies do not include the questionnaire in their paper. Therefore, we focus on the qualitative aspects of participants’ responses with respect to user experience, in short, *what do participants want?*

Some studies did not include any feedback from participants in relation to their experience with the intervention (Rodgers et al., 2019; Khan and Pea, 2017; Wiljer et al., 2020; Kajitani et al., 2020) while others included feedback but not in relation to how the online intervention could be improved (Hookham et al., 2016; Costa et al., 2016). For ThoughtSpot (Wiljer et al., 2020), the authors report using a usability questionnaire with open-ended questions in their study, however, the results of that survey were recently published in a separate research article on March 26, 2021. We include the details of that study in the current analysis (Shi et al., 2021).

Table 6 presents the summarized needs of the participants (students) in these interventions.

Two themes that highlight the importance of including end-users in the design process are “changing the format of the intervention” and “changing the intervention”, it is clear that if these particular students had been included in the development of the intervention, the emergence of such themes would be unlikely.

On the other hand, it is interesting to see that “Access to Professionals” is not mentioned as often as other themes observed in the studies, even though studies like Silvercloud-MindBalance (Doherty et al., 2012) are based on improving adherence through human input. Also, online mental health interventions are usually presented as a more convenient way for students to seek help than face-to-face interventions. Therefore, it is not surprising that students in (Doherty et al., 2012; Farrer et al., 2020; Morr et al., 2020) view them as a convenient and flexible way of receiving help. The digital savviness of this population is frequently mentioned as a reason for proposing these interventions, however, it should be noted that students are fully aware of some of the drawbacks surrounding the use of online interventions. Under the theme “Ensure anonymity/privacy/safety”, we observe that students are highly concerned about who has access to their data. In addition, despite a strong need for “Peer engagement” across the studies, there is a corresponding need for assurance that there would be moderators available to control issues like cyberbullying or negative emotion contagion.

Further, under the theme “Improving interface/presentation”, an example of students’ feedback was “use non-patronizing/non-judgmental language” (Morr et al., 2020). This comment serves as a reminder to researchers that some students have experience with mental health apps/programs, therefore, when designing an online mental health intervention with students, researchers should take advantage of the student's prior experience with such interventions. This could help design interventions that are better tailored to them.

Furthermore, personalization in mental health interventions is not a need that is unique to the student population. A recent survey on users of mental health apps in the general population revealed that users reported getting bored and wanted apps that were tailored to their individual needs (Stawarz et al., 2019).

Moreover, the theme “Make it more engaging”, implies that students have a genuine interest in utilizing these interventions, and it is likely that if they found the intervention to be interesting enough, they may engage with the intervention in the long term. Some example comments from students include “Make it engaging and fun”(Coughlan et al., 2019), “Add regular content updates” (Farrer et al., 2020) etc. Similarly, the theme “Improve Content” shows that students interact sufficiently enough to suggest improvements to the content of the interventions (Kim et al., 2020; Huang et al., 2015; Ananthabhotla et al., 2017; Hickie et al., 2019; Meng et al., 2018; Farrer et al., 2020; Shi et al., 2021).

#### 3.5.4 Defining success

As can be seen from our discussion on user engagement and acceptance, the definition of engagement is varied across the studies. Also, some studies were carried out over short periods of time, therefore, user engagement (defined as participation in the last of week of the intervention) cannot be accurately measured. In other cases, financial incentive is given to participants, making it difficult to measure the true level of engagement. This left us with two studies MindBalance (Sharry et al., 2013) and MePlusMe (Papadatou-Pastou et al., 2019) which were carried out over a period of 8 weeks. We studied these interventions in detail to understand how the differences in the interventions may have contributed to different levels of user engagement. We posit that peer engagement, more stages in the development process, and access to professionals are factors that may have contributed to lower rates of attrition in MindBalance (Doherty et al., 2012).

Conversely, we have discussed the qualitative aspects of user acceptance in all the studies because we believe they serve as a good starting point for researchers attempting to develop online mental health interventions for this population. However, we find it impractical to quantify user acceptance as a measure of success in these interventions. For instance, in MindBalance (Doherty et al., 2012), one participant mentions that the intervention should be changed from an online intervention to a face-to-face intervention. In addition, in UniVirtualClinic (Gulliver et al., 2015), the authors attempt to understand the needs of end-users regarding privacy. Some participants mention their need to have a completely anonymous account while others did not mind having their names associated with their account. Further, in EmotionMap (Huang et al., 2015), one participant mentions that they do not like to focus on their past, while other participants noted that they liked to look at their past emotions as an encouragement to themselves. We observe these dichotomies in participant needs across all studies. Therefore, we posit that user acceptance is not a good measure in defining the success of these interventions.

Moreover, in attempting to answer the research question *How successful are the design methodologies in terms of user acceptance and engagement?*, we find that the answer is not easily addressed. With regards to engagement, we have two interventions that we studied in more detail in order to understand why their engagement differs, however, no definite conclusions can be made from that as there may be other variables present that we do not take into account. On the other hand, we cannot rely solely on the comments from participants as a criterion for the success of these interventions.

## 4 Conclusion and key lessons

### 4.1 Conclusion

In this scoping review, we have reviewed 23 studies (and 19 unique interventions) in which online mental health interventions were designed for students in higher education, while applying user-centered approaches. We have focused on studies that have targeted the improvement of depression symptoms, anxiety symptoms and overall wellbeing. We find it noteworthy that a majority of the interventions included a contextual inquiry in the development of their intervention. This highlights the recognition of the importance of including stakeholders throughout the development process of an intervention.

Further, we found that a majority of the studies included multiple stakeholders in the development process, this is an important aspect of many eHealth development frameworks (van Gemert-Pijnen et al., 2011). Although, only three (Currie et al., 2010; Wiljer et al., 2020; Hickie et al., 2019) of the included interventions mention using a design framework, we note that the included interventions adhere to principles of participatory design (i.e. carrying out a literature review, conducting a contextual inquiry, obtaining user feedback on design concepts etc.).

In an attempt to understand the success of the applied design methodologies in the interventions, we focus on attrition rates and user acceptance. We found that we could only consider two studies - MePlusMe (Papadatou-Pastou et al., 2019) and SilverCloud-MindBalance (Doherty et al., 2012). These interventions were tested by students over a period of 8 weeks in a pilot study and clinical trial, respectively. We posited that peer engagement, access to professionals and more stages in the development process were factors that may have contributed to lower drop-out rates in SilverCloud-MindBalance (Doherty et al., 2012). In addition, in our analysis of user acceptance across the included studies, we performed a qualitative synthesis of themes representing the question “*what students want?”*. The themes identified include convenience, personalization, improved user interface, changes to the format of the intervention, improved content, ensure anonymity/privacy/safety, add peer engagement, include access to professionals, and make it more engaging. Although, these themes are interesting to researchers developing an online mental health intervention for students in higher education, they are not representative outcome of the success of these interventions.

Consequently, we recognize a need for further research on the impact of user-centered design practices on the success of digital mental health interventions for students in higher education. This can only be achieved through the further development of digital mental health interventions, going beyond pilot studies to clinical trials and real world adoption in higher education settings.

### 4.2 Key lessons

In this section, we discuss the key lessons we have learned in our review of user experience in the included studies. We believe these lessons would help researchers attempting to develop online mental health interventions for students in higher education. Although, we could not assess the impact of user centered design practices on the success of digital mental health interventions for students in higher education, other studies on digital mental health interventions in the general population have highlighted the need for user-centered design approaches (Borghouts et al., 2021; Torous et al., 2018).

#### 4.2.1 Fundamental elements

Based on our review of these studies, we prepared a list of fundamental elements that every online mental health intervention should have. They include:

1. Convenience - The survey by the Union of Students in Ireland (Price and Smith, 2019) found that students often complained about the opening hours of the counseling service centres, stating that they could not attend appointments as a result of other responsibilities including work and classes. In addition, in (Doherty et al., 2012; Farrer et al., 2020; Morr et al., 2020), students express the need to have a flexible intervention that could easily fit into their schedule. Therefore, any online intervention designed for students should be flexible and convenient.
2. Personalization - As mentioned earlier, lack of personalization is one of the reasons for low user engagement in online mental health interventions (Stawarz et al., 2019). In addressing user acceptance themes across studies, we also found that personalization was a strong preference for students (Huang et al., 2015; Hickie et al., 2019; Meng et al., 2018; Farrer et al., 2020; Morr et al., 2020), therefore, we posit that researchers should consider personalization in the development of their programs.
3. Anonymous, private and safe - As important as these issues are in any online application, they are seldom mentioned in the included interventions. Our analysis revealed that these issues are important to students. They want to know what happens to their data. They want privacy policies to be written in clear and short forms. They want to be anonymous/named by their choice. Lastly, they want safety to be ensured when interacting with their peers (Gulliver et al., 2015). These issues cannot be ignored in the development of these interventions, as they can deter users from participating in such interventions. For instance, in Gloomy (Kim et al., 2020), one participant did not interact with the program until they understood who could view their data. Also, in UniVirtualClinic(Gulliver et al., 2015), participants’ were concerned about having to register for the online intervention, with one participant stating “You don't want other people to see when you are online, they would say — oh she must be going through something.”
4. Language - In (Morr et al., 2020), participants wanted the use of non-patronizing, non-judgmental language in the online program. Language forms a significant aspect of every online mental health intervention (Khan and Pea, 2017), therefore, to ensure the use of appropriate language, it may be important for researchers to work with stakeholders with experience in the area of mental health support, for example, counselors or academic staff in the field of psychology (van Gemert-Pijnen et al., 2011).

#### 4.2.2 Pay attention to user subgroups

It is important to include end-users in the development of an online mental health intervention, however, we have seen that even in the student population, students have diverse needs. When considering the privacy needs (Gulliver et al., 2015) of students, the authors found that some students desired to have an anonymous account while other students wanted their names associated with their accounts. This is a theme we see across all studies where it seems that students are on opposite sides of the same issue. Therefore, we posit that in the design of an intervention, researchers should seek to understand the diverse subgroups that exist in their student population and design for them. One way of carrying this out in the area of user-centered design is through the use of personas - fictional characters developed through interview, focus groups, etc., which represent the characteristics and needs of particular groups of users (Hickie et al., 2019).

#### 4.2.3 Include end-users throughout

Based on the qualitative user experience garnered across the included studies, we find that most of the feedback would have been avoided if students were included in the development of the interventions.

In their frameworks for eHealth development (Hagen et al., 2012; van Gemert-Pijnen et al., 2011), the authors note that the inclusion of end-users and stakeholders throughout the development of the interventions would lead to fewer errors^5^ and better adherence to the program. Online interventions should be developed with stakeholders throughout the development process, from contextual inquiries to building the prototypes, to pilot studies and so on up to the clinical trials. For instance, FeelingBetter (Currie et al., 2010) applied an iterative usability testing framework (Kushniruk, 2002) through which they refined their program in cycles based on user/stakeholder input. This level of user involvement follows the guideline in the framework (van Gemert-Pijnen et al., 2011) and it could lead to a better user experience and engagement with the intervention.

However, the inclusion of stakeholders (including end-users) should not be perceived as a “magic bullet”. Although participatory design has been found to produce a better user experience, there are challenges associated with designing along with stakeholders. For instance, a diversity of views could exist between and within users and stakeholders, and the researcher will have to decide which to prioritize (Farrer et al., 2020). Also, participatory design is a time-consuming process (Farrer et al., 2020). In addition, implementing the solutions desired by participants can be expensive (Farrer et al., 2020).

Nonetheless, based on our review of included studies, the inclusion of stakeholders in the development of the intervention would create interventions that better reflect the needs, values and context of the end-users.

#### 4.2.4 Additional elements

Peer engagement and access to professionals are other elements that could be added to an online mental health intervention. Although we note that not all research projects would have the resources required to provide these elements. Moreover, peer engagement must include moderators to prevent issues like negative emotion contagion, cyberbullying, etc. (Doherty et al., 2012). In addition, it is commonly known that the counseling centres in universities are overwhelmed by a high number of students seeking help, therefore, the inclusion of those counselors in online mental health intervention could potentially increase their workload, as noted by the counselors in the ISee intervention (Meng et al., 2018). Nonetheless, if feasible, researchers could include these elements, as they may contribute to better user experience and engagement with the intervention.

## Declaration of competing interest

The authors declare that they have no known competing financial interests or personal relationships that could have appeared to influence the work reported in this paper.

## Appendix A Supplementary data

Databases and Search Keywords Image 1

## Footnotes

1 Adherence means using an intervention as intended, for example, completing all exercises in an intervention (Kelders et al., 2012)

2 Attrition means stopping the usage of an online intervention (Eysenbach, 2005)

3 Low user engagement means poor adherence to an intervention (Torous et al., 2018)

4 Engagement in this context means inclusion of end users in the design process of the intervention

5 A closer match between the stakeholders’ needs and the developed intervention
