# Supplementary material for: Online mental health interventions designed for students in higher education: A user-centered perspective
Source: Internet Interv. 2021 Oct 9;26:100468. doi: 10.1016/j.invent.2021.100468 (PMC8524143; doi:10.1016/j.invent.2021.100468)
Supplement: Supplementary file 1 — Databases and Search Keywords [file mmc1.pdf]

Table 1: Databases and Search Keywords

**Database Name**	**Date of Search**	**Search Terms**
IEEE Xplore	13/01/2021	(Design<br>OR “user-centered design”<br>OR “user-centered design”<br>OR “participatory design”<br>OR “co-design”)<br><br>AND (web OR iCBT OR CCBT OR online<br>OR app<br>OR “mobile-app”<br>OR “web-based”<br>OR ehealth OR mhealth OR mobile<br>OR internet OR “e-mental health”)<br><br>AND (Mental health OR wellbeing<br>OR depression<br>OR anxiety OR Mental Disorders)<br><br>AND (“Higher education” OR ”third-level”<br>OR university OR Universities<br>OR undergraduate OR postgraduate<br>OR graduate<br>OR students OR “first-year”<br>OR “college students”<br>OR “university students”)

Table 2: Databases and Search Keywords (contd.)

**Database Name**	**Date of Search**	**Search Terms**
ACM Digital Library	13/01/2021	Abstract:(Design<br>OR “user-centred design”<br>OR “user-centered design”<br>OR “participatory design”<br>OR “co-design”)<br><br>AND Abstract:(“Higher education”<br>OR ”third-level”<br>OR university OR Universities OR undergraduate<br>OR postgraduate OR graduate OR students<br>OR “first-year”<br>OR “college students” OR “university students”)<br><br>AND Abstract:(“Mental health” OR wellbeing<br>OR depression OR anxiety<br>OR “Mental Disorders”)<br><br>AND Abstract:(web OR iCBT<br>OR CCBT OR online OR app OR “mobile-app”<br>OR “web-based”<br>OR ehealth OR mhealth<br>OR mobile OR internet OR “e-mental health”)

Table 3: Databases and Search Keywords (contd.)

**Database Name**	**Date of Search**	**Search Terms**
Web of Science	13/01/2021	#4 AND #3 AND #2 AND #1<br><br>#4(TS = (“Higher education” OR ”third-level”<br>OR universi* OR *graduate<br>OR student?<br>OR “first-year” OR “college student?”<br>OR “university student?”))<br>AND LANGUAGE: (English)<br><br>#3(TS=(Mental health OR wellbeing<br>OR depression OR anxiety<br>OR “Mental Disorders”))<br>AND LANGUAGE: (English)<br><br>#2(TS=(web OR *CBT OR online OR app<br>OR “mobile-app”<br>OR “web-based” OR ehealth OR mhealth<br>OR mobile OR internet* OR “e-mental health”))<br>AND LANGUAGE: (English)<br><br>#1(TS=(“user-cent*d design”<br>OR “participatory design” OR “co-design”))<br>AND LANGUAGE: (English)

Table 4: Databases and Search Keywords (contd.)

**Database Name**	**Date of Search**	**Search Terms**
SCOPUS	13/01/2021	TITLE-ABS-KEY (”Higher education”<br>OR ”third-level” OR universi*<br>OR *graduate<br>OR student? OR ”first-year”<br>OR ”college student?”<br>OR ”university student?”)<br><br>AND TITLE-ABS-KEY (mental AND health<br>OR wellbeing OR depression<br>OR anxiety OR ”Mental Disorder?”)<br><br>AND TITLE-ABS-KEY (web OR ?cbt<br>OR online OR app? OR ”mobile-app”<br>OR ”web-based”<br>OR ?health OR mobile<br>OR internet OR ”e-mental health”)<br><br>AND TITLE-ABS-KEY (”user-cent*d design”<br>OR ”participatory design”<br>OR ”co?design”)

Table 5: Databases and Search Keywords (contd.)

**Database Name**	**Date of Search**	**Search Terms**
Embase	14/01/2021	(design:ti OR ’user-centred design’:ti<br>OR ’user-centered design’:ti<br>OR ’participatory design’:ti<br>OR ’co-design’:ti)<br><br>AND (web:ab OR icbt:ab<br>OR ccbt:ab<br>OR online:ab OR app:ab OR ’mobile-app’:ab<br>OR ’web-based’:ab<br>OR ehealth:ab OR mhealth:ab<br>OR mobile:ab OR internet:ab<br>OR ’e mental health’:ab)<br><br>AND (’mental health’:ab<br>OR wellbeing:ab OR depression:ab<br>OR anxiety:ab OR ’mental disorders’:ab)<br><br>AND (’higher education’:ab<br>OR ’third-level’:ab OR university:ab<br>OR universities:ab OR undergraduate:ab<br>OR postgraduate:ab<br>OR graduate:ab OR students:ab<br>OR ’first-year’:ab<br>OR ’college students’:ab<br>OR ’university students’:ab)

Table 6: Databases and Search Keywords (contd.)

Database Name	Date of Search	Search Terms
Google Scholar	14/01/2021	(Design OR “user-centred design”<br>OR “user-centered design”<br>OR “participatory design” OR “co-design”)<br><br>AND (web OR iCBT OR CCBT<br>OR online OR app<br>OR “mobile-app” OR “web-based”<br>OR ehealth<br>OR mhealth OR mobile<br>OR internet<br>OR “e-mental health”)<br><br>AND (“Mental health”<br>OR wellbeing OR depression OR anxiety<br>OR “Mental Disorders”)<br><br>AND (“Higher education”<br>OR ”third-level” OR university OR Universities<br>OR undergraduate<br>OR postgraduate<br>OR graduate OR students<br>OR “first-year” OR “college students”<br>OR “university students”)

Table 7: Databases and Search Keywords (contd.)

Database Name	Date of Search	Search Terms
EBSCO Host	14/01/2021	TI (Design OR “user-centred design”<br>OR “user-centered design”<br>OR “participatory design” OR “co-design”)<br><br>AND AB (web OR iCBT<br>OR CCBT OR online OR app<br>OR “mobile-app” OR “web-based”<br>OR ehealth OR mhealth<br>OR mobile OR internet<br>OR “e-mental health”)<br><br>AND AB (Mental health OR wellbeing<br>OR depression OR anxiety<br>OR Mental Disorders)<br><br>AND AB (“Higher education”<br>OR ”third-level” OR university<br>OR Universities OR undergraduate<br>OR postgraduate<br>OR graduate<br>OR students OR “first-year”<br>OR “college students”<br>OR “university students”)

Table 8: Databases and Search Keywords (contd.)

Database Name	Date of Search	Search Terms
Pubmed	14/01/2021	(((Design[Title] OR "user-centred design"[Title]<br>OR "user-centered design"[Title]<br>OR "participatory design"[Title]<br>OR "co-design"[Title])<br><br>AND (web[Title/Abstract]<br>OR iCBT[Title/Abstract]<br>OR CCBT[Title/Abstract]<br>OR online[Title/Abstract]<br>OR app[Title/Abstract]<br>OR "mobile-app"[Title/Abstract]<br>OR "web-based"[Title/Abstract]<br>OR ehealth[Title/Abstract]<br>OR mhealth[Title/Abstract]<br>OR mobile[Title/Abstract]<br>OR internet[Title/Abstract]<br>OR "e-mental health"[Title/Abstract]))<br><br>AND (Mental health[Title/Abstract]<br>OR wellbeing[Title/Abstract]<br>OR depression[Title/Abstract]<br>OR anxiety[Title/Abstract]<br>OR Mental Disorders[Title/Abstract]))<br><br>AND ("Higher education"[Title/Abstract]<br>OR "third-level"[Title/Abstract]<br>OR university[Title/Abstract]<br>OR Universities[Title/Abstract]<br>OR undergraduate[Title/Abstract]<br>OR postgraduate[Title/Abstract]<br>OR graduate[Title/Abstract]<br>OR students[Title/Abstract]<br>OR "first-year"[Title/Abstract]<br>OR "college students"[Title/Abstract]<br>OR "university students"[Title/Abstract])
